# Nutritional value and prediction of digestible and metabolizable energy of full-fat deactivated soybeans for pigs

**DOI:** 10.5713/ab.23.0519

**Published:** 2024-06-27

**Authors:** Teresinha Marisa Bertol, Jorge Vitor Ludke, Arlei Coldebella, Herbert Rech

**Affiliations:** 1 Embrapa Suínos e Aves, BR 153, KM 110, Vila Tamanduá, Concórdia, SC, 89715-899, Brazil; 2 Department of Animal Science, Universidade Federal do Rio Grande do Sul, Av. Bento Gonçalves, 776 Bairro Agronomia, Porto Alegre, RS, 91540-000, Brazil

**Keywords:** Digestibility, Full-fat Deactivated Soybeans, Metabolizable Energy, Pigs, Processing Parameters

## Abstract

**Objective:**

The objective of this work was to determine the energetic values of 14 full-fat deactivated soybeans samples, the effect of partial removal of the hull, and to develop equations for predicting digestible (DE), metabolizable (ME), and ME corrected for nitrogen balance (ME_n_) for pigs.

**Methods:**

Ten metabolism experiments were conducted over a two-year period to evaluate 14 batches of full-fat deactivated soybeans, following the method of the total collection of feces and urine. One hundred and ninety-two pigs with an average initial body weight of 51.4±5.4 kg were assigned to dietary treatments.

**Results:**

Partial dehulling of soybeans did not affect DE, ME, and ME_n_ values. The variables that best explained the variations (p<0.05) in DE were ureatic activity (UA) and crude fiber. The variables that showed the greatest association (p<0.05) with ME and ME_n_ were UA, protein solubility, and processing pressure. The observed effect of UA on energy values was quadratic (p<0.05). Phosphorus also showed association (p<0.05) with DE and ME and the energy applied per kg of sample showed association (p<0.05) with ME and ME_n_.

**Conclusion:**

The overall mean values of DE, ME, and ME_n_ were 4,558, 4,457, and 4,344 kcal/kg, respectively. The partial removal of the hull prior to soy deactivation did not affect the digestibility or the energy values. This study shows that the processing conditions are the main factors affecting the energetic value of full-fat deactivated soybeans for pigs, which can be accurately predicted using a combination of chemical composition, quality indicators, and processing parameters.

## INTRODUCTION

Soybean is the main protein supplier ingredient for pig production in Brazil. However, raw soybeans contain antinutritional factors that can negatively affect pig growth performance. The main antinutritional factors found in soybeans are lectins and protease inhibitors, but these can be inactivated by thermal processing [1], thus increasing the digestibility of the nutrients and energy. Several processing methods have been proposed to inactivate full-fat soybeans, such as wet or dry extrusion, micronization, wet or dry roasting, jet-sploding, cooking, and microwaving. Different equipment and variations in the processing parameters may cause high variability in the digestibility and energy values of full-fat soybeans, as can be observed in the previous studies [2–7]. The optimal method depends on the cost of processing and final product quality, that is, the ability to inactivate the antinutritional factors without impairing the digestibility of components.

The process of deactivating anti-nutritional factors in full-fat soybeans using hermetic reactors under controlled conditions of steam injection, temperature, and pressure is widely used in Brazil. This process applied to whole grains involves the application of steam under a temperature between 63°C and 107°C and pressure of 4 to 8 kgf/cm^2^ and in a vacuum [8]. However, the processing parameters may vary among different industrial plants or even within the same plant, resulting in variable quality of the final product. In addition, variations in the moisture content, chemical composition and content of antinutritional factors of raw soybeans may influence the nutritional value of the deactivated soybeans. Another variation factor is the proportion of hulls in the grains as some industries partially peel the grains before processing. Ludke et al [4] observed a variation of 330 kcal/kg in the metabolizable energy (ME) for pigs, in three samples of full-fat deactivated soybeans obtained with different equipment and processing parameters. Toledo et al [9] reported a 343 kcal/kg ME difference between unpeeled and dehulled full-fat deactivated soybeans. Nunes et al [6] found a 953 kcal/kg ME difference for broilers among eight samples of full-fat deactivated soybeans, which the authors credited to variations in the ether extract (EE) and fiber content of the samples. Considering this variability, applying mean energy values of processed full-fat soybeans for diet formulation may not be adequate. Furthermore, no nutritional values are reported in the main tables of feed composition for pigs specific for full-fat deactivated soybeans processed with the hermetic reactors as described above. Given the paucity of information about this product and the variable energy values reported in different studies, the objectives of this study were to determine the digestible energy (DE), ME, and ME corrected for nitrogen balance (ME_n_) of 14 samples of unpeeled and partially dehulled full-fat deactivated soybeans processed with hermetic reactors and to develop equations based on chemical composition, quality indicators, and processing parameters to predict DE, ME, and ME_n_.

## MATERIALS AND METHODS

The research was approved by the Animal Research Ethics Committee (CEUA/CNPSA) under protocol number 018/2018, following the Ethical Principles in Animal Experimentation (CFMV Resolution 879, 2008) adopted by the Brazilian College of Animal Experimentation (COBEA) and in accordance with the technical guidance of CONCEA no. 8 of March 18, 2016.

### Samples and processing

Fourteen samples of full-fat deactivated soybeans were collected from five industrial soy processing plants located in the states of Rio Grande do Sul, Santa Catarina, São Paulo, and Tocantins, seven of which were unpeeled samples and seven partially dehulled samples. The samples were processed using hermetic reactors under controlled steam injection, temperature, and pressure conditions. The processing parameters varied among plants (Table 1). The samples were ground in a hammer mill using a screen with an aperture of 3.5 mm for inclusion in the experimental diets.

The amount of energy applied per kg of sample (EAS) was calculated using the processing parameters and the specific heat (SH) of samples [10], as follows:


$$\begin{array}{l} \text{SH }(\text{kJ}/\text{kg})=0.391+0.461\;[\text{M}/(100+\text{M})]\\ \text{SH }(\text{cal}/\text{kg})=\text{SH }(\text{kJ}/\text{kg})\times 4.1868\times 1,000\\ \text{M}=\%\;\text{moisture of soybean}\\ \text{EAS }(\text{cal}/\text{kg})=\text{SH}\times \text{TIM}\times \text{TPT}\\ \text{EAS }(\text{Mcal}/\text{kg})=\text{EAP}/1,000,000\\ \text{TPT }({}^{\circ}\text{C})=\text{processing temperature}\\ \text{TIM }(\text{min})=\text{processing time} \end{array}$$

### Digestibility and metabolism

Metabolism experiments followed the methodology of total collection of feces and urine as described by Sakomura and Rostagno [11]. The reference diet (RD) was present in every assay. Deactivated soybeans replaced 30% of the RD in the test diets (TD). The RD was formulated to meet the nutritional requirements of barrows with a live weight of 50 to 70 kg [12] (Table 2). Ten experiments were conducted, with the evaluation of 1 to 3 soybean samples in each experiment, resulting in a total of 10 RD and 14 TD. Eight pigs (replications) were used per treatment, with a total of 192 pigs (average initial weight = 51.4±5.4 kg), housed in individual metabolic cages [13]. Pigs were allotted to the treatments according to initial weight (block) in a randomized block design. The first 7 days of the experiment were for the pig’s adaptation to the cages and diets and to determine the daily feed intake (DFI). Thereafter followed five days of feces and urine collection. The individual voluntary DFI in the adaptation period was recorded for each block and used to determine the daily amount of feed offered in the collection period as follows: the constant of metabolic weight (CMW; body weight^0.75^) of the lightest pig in each block was calculated (CMW = feed intake/metabolic weight), that is, grams of feed per kg of metabolic weight; subsequently, the daily amount of feed offered to each pig was obtained by multiplying the metabolic weight of each pig by the CMW (daily feed = CMW× metabolic weight). Therefore, all pigs in the same block received the same amount of feed per kg of metabolic weight. The daily amount was divided into two meals, offered at 07:30 and 14:00. Water was offered *ad libitum* after each meal. Ferric oxide was used as a fecal marker in the proportion of 0.5%, to indicate the beginning and the end of the fecal collection. Feces were collected twice a day and stored in plastic bags. The urine was collected in plastic buckets containing a 20 mL 1:1 solution of distilled water and concentrated HCl (reagent grade, 12 *N*). Every morning, the volume of urine collected was measured and an aliquot of 20% was obtained and stored in refrigerator. Feces were stored in a freezer at −8°C. At the end of the collection period, the pool of feces and urine from each experimental unit was homogenized and a sample of each one was collected. The feces samples were dried in a forced-air oven at 55°C for 72 h and ground in a knife mill (Willey) with a 1-mm aperture strainer. Nitrogen balance, coefficients of apparent digestibility of dry matter (DM) and crude protein (CP), and the biological value of protein, DE, ME, and ME_n_ values were calculated according to the equations proposed by Matterson et al [14].

### Sample analysis

Soybean samples were analyzed for DM content (Method 934.01), ash (Method 942.05), CP as total N by Dumas (Method 990.03) and the result multiplied by 6.25, crude fiber (CF; Method 962.09) according to the procedures of the Association of Official Analytical Chemists (AOAC) [15]. Ether extract was analyzed with the automated extractor Ankon XT-15 (ANKOM Technology, Macedon, NY, USA) according to AOCS [16], acid detergent fiber (ADF) and neutral detergent fiber (NDF) according to Van Soest et al [17], gross energy (GE) by calorimetry (Leco Corporation, St Joseph, MI, USA). Calcium and phosphorus by spectrometry of optic emission with inductively coupled plasma (ICP OES Optima 8300; Perkin Elmer, Waltham, MA, USA) and phytic phosphorus (phytic acid)/total phosphorus measured as phosphorus released by phytase and alkaline phosphatase “K-PHYT 08/14”, Megazyme International Ireland, 2014. Ureatic activity (UA) was measured through the pH variation (ΔpH) as a function of the ammonia released by the enzymatic action of urease (Method Ba 9–58) [18]. Protein solubility (PS) was determined as soluble protein in KOH 0.036 M solution and posterior quantification by the Kjeldahl method [19]. Conventional and sulphur amino acids were analyzed by liquid chromatography, with pre-column derivatisation with phenyl isothicyanate [20,21]. Tryptophan was analyzed according to Lucas and Sotelo [22]. Diets and feces were analyzed for DM, CP, and GE as described for soybean samples. Urine was analyzed for N (Method 984.13) [15] and GE. For GE analysis, an aliquot of urine (5 mL) was transferred to polystyrene beakers (Fisherbrand P/N 08–732–119), dried in a forced-air oven at 50°C for 24 h and the GE determined using the same bomb calorimeter used for the soybean samples.

### Statistical analysis and equation modelling

Data from unpeeled and partially dehulled samples were compared by t-test for unpaired data, using the TTEST procedure of SAS (version 9.4; SAS Institute, Cary, NC, USA). For composition and GE data, quality indicators and processing parameters, the sample was considered as the experimental unit. For DE, ME, and ME_n_, the experimental unit considered was the average of each experiment. Differences were considered significant at p<0.05. Correlation analysis between DE, ME, and ME_n_ with chemical composition, quality indicators (UA and PS) and processing parameters was performed using the CORR procedure of SAS.

Prediction equations for DE, ME, and ME_n_ were developed using the GENMOD procedure of SAS. Chemical composition, GE, quality indicators, processing parameters and EAS data of deactivated soybean samples were used as predictor variables for the development of the equations. These variables were included as linear components, which, combined with some interactions of interest, allowed access to 82 possible linear models for DE, 329 linear models for ME, and 242 linear models for ME_n_. It was assumed that DE, ME, and ME_n_ have a normal distribution. The maximum likelihood method was used to estimate the model parameters. The best model choice was based on the Akaike information criterion (AIC), for which the coefficient of determination and the prediction errors (absolute and relative) were calculated.

## RESULTS

### Chemical composition, quality indicators and energy values

The average DFI of RD was similar to TD (1,702 vs 1,716 g/d; Table 3). The average CP content of TD was about six percentual points higher than the average CP of RD (24.22% vs 18.17%). Besides the difference in protein content between the RD and TD, the ME/DE ratio and ME_n_/DE ratio are similar between them. Digestible energy, ME, and ME_n_ showed wide variation among the soybean samples (Table 4). The chemical components with the highest variation among the samples are CF, ADF, and NDF. The CF, NDF, and ADF contents were higher (p<0.05) in unpeeled than in partially dehulled deactivated soybeans, however, there was no difference in DE, ME, and ME_n_ values between the two groups (Table 5).

### Amino acids

The greatest relative variation in amino acid content between samples was observed in hydroxyproline (23.19%), cystine (12.73%), and Isoleucine (8.12%; Table 6). There was no difference in the amino acid content between the unpeeled and partially dehulled soybeans, except for alanine, which had a higher content (p<0.05) in the partially dehulled samples (Table 7).

### Correlations and modelling of prediction equations

Processing pressure (PP) was negatively correlated (p<0.05) with DE, ME, and ME_n_ (r = −0.53, −0.59, and −0.57, respectively), while CF was negatively correlated (p<0.05) with DE (r = −0.65; Table 8). On the other hand, phosphorus was positively correlated (p<0.05) with DE, ME, and ME_n_ (r = 0.54, 0.53, and 0.56, respectively) and PS was positively correlated (p<0.05) with ME and ME_n_ (r = 0.55 and 0.61, respectively).

The best equations for DE, ME, and ME_n_ prediction were selected based on AIC value and prediction error. The variables that best explain the variation in DE are UA (R^2^ = 0.69) and CF (R^2^ = 0.42; Table 9). The effect of UA is quadratic (p<0.05), with the maximum DE value standing between 0.041 and 0.046 ΔpH. Equation 1 (AIC = 166.24 and R^2^ = 0.85), based on CF, UA, and phosphorus, is the one that best explains the DE values. According to equation 2 (AIC = 167.51 and R^2^ = 0.84), replacing phosphorus with CP causes a small loss of precision. Equations 3 (AIC = 167.74 and R^2^ = 0.84), based on phosphorus, NDF and UA and 4 (AIC = 168.74 and R^2^ = 0.80) based on CF and UA, are very close in accuracy to equation 2, with small increases in AIC and the prediction error. Of the four equations with the lowest AIC, lowest prediction error and highest R^2^, equations 2 and 4 seem to be the most plausible for practical use because they are based on more measurable variables than equations 1 and 3 which include phosphorus.

Eight equations were selected for ME prediction (Table 10). Ureatic activity, PP, and PS explain the 54%, 35%, and 30%, respectively, of the variation in ME. The effect of UA is quadratic (p<0.05) and the UA range within which the highest ME value lies is between 0.044 to 0.055 ΔpH. The best equation includes PS, PP, UA, and EAS as predictor variables (AIC = 176.69 and R^2^ = 0.80). Excluding PS and EAS and including ash and CF results in a small loss of precision (AIC = 178.59 and R^2^ = 0.77). Among the 8 selected equations, equations 2 and 4 are the best for practical use because are based solely on quality indicators and chemical composition variables.

**Figure 1:**
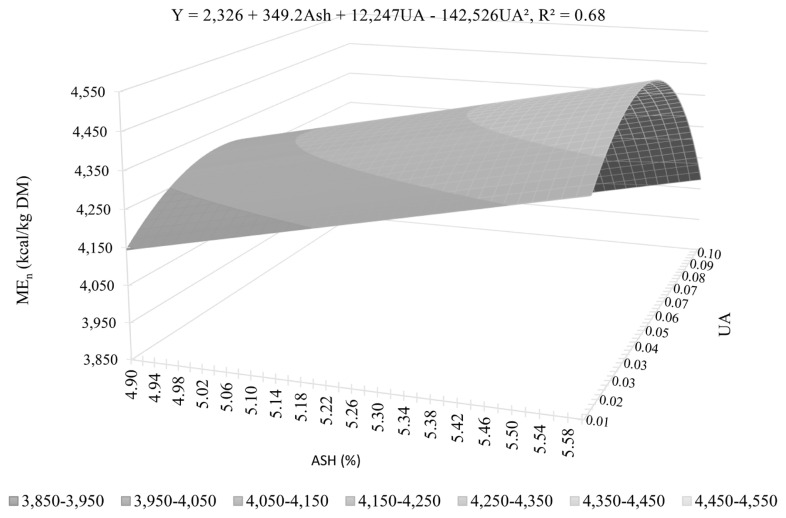
Predicted values and maximum point of metabolizable energy corrected for nitrogen balance (ME_n_) of full-fat deactivated soybeans for pigs according to the prediction equation based on ureatic activity (UA) and Ash (dry matter basis).

The variation in ME_n_ was mainly explained by UA (R^2^ = 0.54), PS (R^2^ = 0.37), and PP (R^2^ = 0.33; Table 11). Other variables, such as EAS, CF, and ash, also showed an effect (p<0.05) on the estimated maximum likelihood parameters. As in the DE and ME, UA has a quadratic shape as shown in Figures 1 (equation 5) and 2 (equation 6) and is individually the variable that best explains the ME_n_ values. A quadratic effect was observed for UA, with the highest ME_n_ value found between 0.043 and 0.055 ΔpH. Equation 1, based on PS, PP, UA and EAS, presents the lowest AIC value and the lowest mean prediction error (AIC = 171.21 and R^2^ = 0.84), and is, therefore, the equation that best estimates the ME_n_ values. Excluding EAS (equation 3) considerably reduces the accuracy of the prediction (AIC = 175.22 and R^2^ = 0.75). The best ME_n_ prediction equation from the set of equations for practical use (equation 2), based on PS, UA, ash and CF, showed a similar level of precision to equation 3 (AIC = 176.11 and R^2^ = 0.77). Excluding CF (equation 4) results in losses in precision, indicated by the increased AIC value (AIC = 177.69) and prediction error and reduced R^2^ (0.71). Equations 6 (AIC = 178.62 and R^2^ = 0.64) based on PS and UA and 7 (AIC = 179.86 and R^2^ = 0.54), based solely on UA, explain 64% and 54% of the variation in ME_n_ values, respectively, and show the importance of the effect of these parameters on the nutritional value of deactivated soybeans.

With a few exceptions, all the predictor variables contemplated in the prediction equations for DE, ME, and ME_n_ showed significant (p<0.05) effect (Table 12).

## DISCUSSION

### Quality indicators and energy values

The UA values lay between 0.01 and 0.10 ΔpH, therefore, some samples were below the industry-standard range for adequate processing (0.03 and 0.16 ΔpH) [23]. Protein solubility varied between 75.1% and 86.2%, therefore, all values lay within the range considered as adequate [24,25]. One of the reasons for the variation observed in these quality indicators is the use of different processing parameters among the different industrial plants. It is important to highlight that two of the samples with the highest UA had a PS of below 80% (sample 10: UA = 0.08, PS = 78.8%; sample 14: UA = 0.10, PS = 78.0%) and one of the samples with the lowest UA had a PS of above 80% (sample 5: UA = 0.01, SP = 84.8%). Ureatic activity and PS showed no correlation (r = −0.28). The content of trypsin inhibitors differs among the different soybean varieties [26], which may contribute to the variation in the UA between different samples of deactivated soybeans, independently of the PS. According to the standards adopted by agroindustry, UA values below 0.03 and PS below 80% indicate over-processing. However, according to Araba and Dale [24], even zero UA may not be indicative of overprocessing and may not be harmful to the nutritional value of the soybeans, with no effect on animal performance. The positive correlation between PS and ME_n_ is probably due to less damage to protein during processing in samples with high PS. However, PS values below 80% do not necessarily indicate damage to the raw material by over-processing at a level that affects nutrient digestibility and animal performance, which was observed only when PS was below 70%. Another important factor is that the values considered as adequate for UA and PS were defined based on soybean meal quality standards. However, different processing methods of full-fat soybeans including heat, pressure and steam could result in more effective deactivation of antinutritional factors and, consequently, lower UA values, without affecting the PS (minimizing the Maillard reaction), which would require new reference standards for UA and PS according to the processing method. In addition, it is important to investigate why there were samples with a low PS (below 80%) among the samples with a high UA (ΔpH 0.08 to 0.10).

The initial moisture content in the grain may interfere with the deactivation of antinutritional factors, as Žilić et al [26] observed in the extrusion process, especially in the wet extrusion. This could be an additional variation factor between different batches of products and different industries. The samples of deactivated soybeans evaluated in this study came from five different plants. Time, temperature and pressure data shown in Table 1 represent the standard configuration used by the processors, however, variations between different batches of the product may occur due to equipment and process monitoring failures, generating under-processed or over-processed batches. It may be inferred that the samples were subjected to process oscillations or were even affected by the variation of soybean inlet moisture, reducing the denaturation potential of antinutritional enzymes, expressed in 3 of the 14 soy samples, which had a UA of between 0.07 and 0.10 (sample 6: plant 4; sample 10: plant 1; sample 14: plant 3). Furthermore, two samples had low UA values (0.01 to 0.02 ΔpH) associated with PS below 80% (samples 4 and 12: plant 4), which may also have been caused by processing failures or poor quality of the raw material.

The difference between the minimum and maximum DE, ME, and ME_n_ values observed in the deactivated soybean samples was 485, 651, and 627 kcal/kg, respectively, which suggests an effect of chemical composition and processing parameters on the nutritional value of the soy.

The constancy of the energy values between samples of unpeeled and dehulled deactivated soybeans indicates that other factors exerted greater influence on the variation of energy values than the presence or absence of hulls. On the other hand, Toledo et al [9] reported a difference of 343 kcal ME between unpeeled and dehulled full-fat deactivated soybean samples. The average DE and ME_n_ values obtained for partially dehulled deactivated soybeans are similar to those reported by Toledo et al [9], however, for unpeeled soybeans, DE and ME_n_ were superior in this study compared to the values obtained by those authors. This difference is probably due to the fact that Toledo et al [9] worked with nursery piglets, which have less capacity to digest some components of the diet, especially fibers, compared to growing pigs.

The results of this study corroborate the reports of other authors regarding the effect of processing parameters on the energy values of full-fat deactivated soybeans. Ludke et al [4] observed a variation of 330 kcal/kg in three samples of soybeans deactivated through different equipment and with variations in processing parameters. Carvalho et al [27] observed higher energy values for vacuum-deactivated soybeans (89.3% PS and 0.12 ΔpH UA) compared to steam-deactivated soybeans (74.5% PS and 0.03 ΔpH UA). Nunes et al [6] observed a 1,048 kcal/kg variation in the ME for broiler chickens among 8 samples of full-fat deactivated soybeans, which was attributed to the lack of standardization in the processing.

The lowest ME and ME_n_ values were observed in two samples with UA between 0.08 and 0.10 ΔpH and PS below 80% (sample 10: ME = 4,297 kcal/kg DM, ME_n_ = 4,166 kcal/kg DM; sample 14: ME = 4,070 kcal/kg DM, ME_n_ = 3,983 kcal/kg DM), one sample with UA of 0.02 ΔpH associated with PS of 75% (sample 12: ME = 4,426 kcal/kg DM, ME_n_ = 4,289 kcal/kg DM), and one sample with UA and PS within the range of adequate processing, but with high CF content (6.2%) (sample 7: ME = 4,313 kcal/kg DM, ME_n_ = 4,230 kcal/kg DM). In addition, one sample with UA = 0.01 ΔpH and PS = 83% showed one of the lowest ME_n_ values (sample 9: ME = 4,217 kcal/kg DM, ME_n_ = 4,155 kcal/kg DM), which could be related to the poor quality of the raw material before processing. These results suggest that for this type of processing, UA values from 0.08 to 0.10 ΔpH negatively affect the energy values of soybeans and corroborate the reports of Milani et al [7], which observed that non-toasted soybean meal with 0.10 UA had lower GE digestibility than non-toasted extruded soybean meal with 0.03 ΔpH. The results of this study are also in accordance with Nunes et al [6], who observed the lowest ME_n_ values of deactivated soybeans in samples with high UA (0.32 ΔpH), low PS (73.3%) or high CF (5.93%).

Some factors that could interfere with the nutritional value of soybeans are tillage practices, delays in harvest, harvesting and post-harvest grain management, as well as storage time, and storage conditions before and after processing for final use. Long storage periods may cause a reduction in the nitrogen solubility index, decreased reducing- and non-reducing-sugars [28], reduction of protein dispersibility index [29] and lipid content [30], and increased oxidation of lipids [31] and tocopherols [32], which may affect the nutritional value of the soybeans regardless of the UA.

The main swine feed composition tables [12,33,34] contain no referenced DE, ME, and ME_n_ values specific for deactivated full-fat soybeans processed by hermetic reactors as described in this study. The average ME value of the deactivated full-fat soybeans obtained in this study (4,457 kcal/kg DM) is higher than the average ME value reported for full-fat soybeans (4,265 kcal/kg DM) in the NRC [33]. For ME_n_, the average value obtained in this study (4,344 kcal/kg DM) is close to the value reported for full-fat extruded soybeans in Brazilian tables for poultry and pigs (4,277 kcal/kg DM) [12].

### Correlations and modelling of prediction equations

Despite being the variable that best explains the variations in DE, ME, and ME_n_, UA failed to show a significant correlation with any of the energy values due to its quadratic behavior as a function of UA.

As the processing parameters are not of practical use by the agroindustry, but explain a large part of the variation in the ME and ME_n_ values, the equations that contemplate these parameters were considered as theoretical and the equations based only on chemical composition and quality indicators were considered as of practical use.

In the literature, DE prediction equations were found only for soybean meal [35,36], but not for deactivated soybeans. Kang et al [35] determined that the best equation for predicting soybean meal DE was based on ash, EE, acid detergent lignin, non-nitrogenous extractive (NNE) and calcium. These authors did not include UA in the set of predictive variables for the modelling. Ellery et al [36] found that the best prediction equation for soybean meal DE was based on CP, EE, ash, CF, ADF, and NDF, but these authors did not include UA and PS in the modelling. Therefore, some of the predictive variables of DE observed in this study differ from those observed by other authors, emphasizing UA.

In the same way as for DE, no ME and ME_n_ prediction equations specific for full-fat deactivated soybeans were found in the literature. Some equations were developed to predict the ME_n_ of soybean by-products for broilers [37] or pigs [35,36] and for a set of protein ingredients for poultry [38], in addition to the equation proposed by Rostagno et al [12] for raw materials of vegetable origin and dairy products for pigs. Only Kang et al [35] included UA and PS as possible predictive variables of ME. Still, these variables did not compose the equations indicated by these authors as the best for predicting ME. The EE was the only common variable in all the equations identified as the best by each of the cited authors, except in those developed by Kang et al [35]. The absence of EE as a predictor variable in the present study is probably due to the low range of variation observed in this component in the evaluated samples. Crude fiber, ADF, NDF, ash, CP, NNE and soluble carbohydrates are also present as predictive variables of ME or ME_n_ in different equations reported by the aforementioned authors. Zonta et al [39], in a validation study of the equations developed by other authors, observed that the equations that best estimated the ME_n_ of extruded, toasted and micronized full-fat soybeans for poultry were those developed by Rodrigues [40] and Rodrigues et al [37], which include the variables CP, ADF and EE or CF, EE, ash and starch. Therefore, the identification of UA and PS as predictors of DE, ME, and ME_n_ in this study differs from all the other cited studies. It is noteworthy that even with the low amplitude observed in the UA values (0.01 to 0.10 ΔpH), it was still possible to detect the strong impact of this factor on DE, ME, and ME_n_.

## CONCLUSION

The DE, ME, and ME_n_ content of full-fat deactivated soybeans ranged from 4,248 to 4,733 kcal/kg DM, 4,070 to 4,721 kcal/kg DM, and 3,983 to 4,610 kcal/kg DM, and the overall mean values were 4,558, 4,457, and 4,344 kcal/kg DM, respectively. The partial removal of the hull prior to soy deactivation did not affect the digestibility or the DE, ME, and ME_n_ values.

The variables that best explain the variations in DE were UA and CF. The processing parameters and quality indicators showed a strong association with ME and ME_n_, consequently UA, PS, and PP were the variables that showed the greatest association with ME and ME_n_. The observed effect of UA on energy values was quadratic. The UA range that maximized DE was between 0.041 and 0.046 ΔpH. Metabolizable energy and ME_n_ showed maximum estimated values of between 0.043 and 0.055 ΔpH.

The results of this study show that the processing conditions are the main factors affecting the energetic value of full-fat deactivated soybeans for pigs, which can be accurately predicted using a combination of chemical composition, quality indicators, and processing parameters. Therefore, the prerequisites for predicting the real energy value of full-fat deactivated soybeans rest on a broader set of parameters that must accompany their identity when this ingredient is intended for pig feeding.

## Figures and Tables

**Figure 2:**
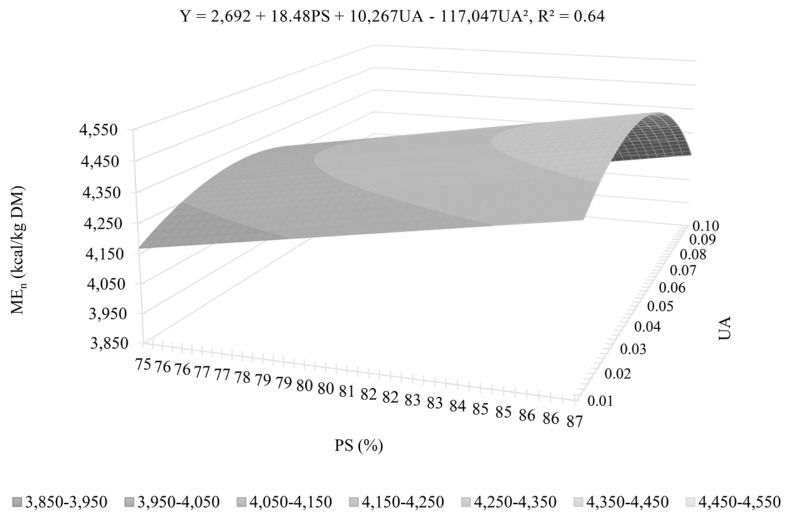
Predicted values and maximum point of metabolizable energy corrected for nitrogen balance (ME_n_) of full-fat deactivated soybeans for pigs according to the prediction equation based on protein solubility (PS) and ureatic activity (UA) (dry matter basis).

**Table 1:** Processing parameters used by the different industrial plants

Variable	Plant 1	Plant 2	Plant 3	Plant 4	Plant 5
No. of samples	1	2	2	6	3
Time (s)	180	960	1500	900	900
Temperature (°C)	115	108	120	120	110
Pressure (kgf/cm^2^)	0.8	1.0	2.0	0.9	0.8
Final moisture (%)	11.1	9.6	11.5	11.4	11.6
Protein solubility (%)	78.8	83.1	80.7	81.4	83.2
Ureatic activity (ΔpH)	0.080	0.035	0.070	0.028	0.033

**Table 2:** Ingredient composition (as-fed basis) of reference diet used in the metabolism experiments

Ingredient	%
Corn	76.291
Soybean meal	20.314
Dicalcium phosphate	1.456
Limestone	0.775
Salt	0.474
L-Lysine HCl 78.8%	0.303
DL-Methionine 99%	0.044
L-Threonine 98%	0.043
Vitamin-mineral premix^1)^	0.300

1) Supplied per kg of diet: Cu, 85.05 mg as copper sulphate; Fe, 90.45 mg as ion sulphate; Zn, 80.55 mg as zinc oxide; Mn, 30.30 mg manganese sulfate; I, 0.83 mg as calcium iodate; Se, 0.20 mg as sodium selenite; vitamin A, 6,750 IU; vitamin D_3_, 1,350 IU; vitamin E 15 IU; vitamin K_3_, 0.90 mg; folic acid, 0.342 mg; pantothenic acid, 9.04 mg; biotin, 0.09 mg; niacin, 16.79 g; vitamin B_1_, 1.01 mg; vitamin B_12_, 16.87 mcg; vitamin B_2_, 2.83 mg; vitamin B_6_, 1.12 mg; choline, 0.05 g; ethoxyquin, 0.75 mg.

**Table 3:** Daily feed intake (DFI, kg/d) and analyzed crude protein (CP, % DM) and energy values (kcal/kg DM) of reference diets (RD) and test diets (TD)

Exp	Diet	DFI	CP	DE	ME	ME_n_	ME/DE	ME_n_/DE
1	RD	1.559	18.36	3,873	3,787	3,695	97.8	95.4
1	TD-Sample 1	1.550	22.99	4,133	4,072	3,974	98.5	96.2
1	TD-Sample 2	1.555	23.66	4,127	4,055	3,953	98.3	95.8
2	RD	1.636	18.56	3,819	3,738	3,656	97.9	95.7
2	TD-Sample 3	1.585	25.26	4,085	4,017	3,910	98.3	95.7
3	RD	1.708	16.75	3,857	3,758	3,683	97.4	95.5
3	TD-Sample 4	1.747	22.97	4,060	3,968	3,875	97.8	95.5
4	RD	1.751	17.61	3,897	3,787	3,702	97.2	95.0
4	TD-Sample 5	1.768	23.27	4,074	3,995	3,908	98.1	95.9
4	TD-Sample 6	1.779	23.62	4,121	4,020	3,931	97.5	95.4
5	RD	1.820	18.39	3,890	3,780	3,701	97.2	95.2
5	TD-Sample 7	1.819	24.35	4,052	3,943	3,863	97.3	95.3
6	RD	1.557	17.55	3,880	3,821	3,730	98.5	96.1
6	TD-Sample 8	1.555	24.02	4,117	4,065	3,963	98.7	96.2
7	RD	1.650	18.83	3,908	3,815	3,727	97.6	95.4
7	TD-Sample 9	1.638	24.43	4,032	3,936	3,856	97.6	95.6
8	RD	1.749	17.78	3,868	3,785	3,700	97.9	95.7
8	TD-Sample 10	1.716	23.82	4,019	3,937	3,838	97.9	95.5
9	RD	1.832	18.15	3,859	3,760	3,681	97.4	95.4
9	TD-Sample 11	1.862	24.90	4,091	3,985	3,890	97.4	95.1
9	TD-Sample 12	1.819	24.83	4,103	3,965	3,868	96.6	94.3
9	TD-Sample 13	1.842	24.86	4,129	3,963	3,881	96.0	94.0
10	RD	1.753	20.22	3,846	3,738	3,647	97.2	94.8
10	TD-Sample 14	1.793	25.36	3,969	3,840	3,750	96.7	94.5

DM, dry matter; Exp, experiment; DE, digestible energy; ME, metabolizable energy; ME_n_, ME corrected for nitrogen balance.

**Table 4:** Descriptive statistics of chemical composition, energy values (dry matter basis) and quality indicators of full-fat deactivated soybean samples

Variable	Mean (n = 14)	SD	Minimum	Maximum
Crude protein (%)	39.97	1.08	38.19	42.50
Ether extract (%)	24.49	1.30	21.02	26.08
Ash (%)	5.32	0.18	4.93	5.56
Crude fiber (%)	4.53	1.06	2.67	6.20
Acid detergent fiber (%)	5.34	1.23	2.88	7.05
Neutral detergent fiber (%)	10.42	1.87	6.42	13.94
Hemicellulose (%)	5.08	1.31	3.55	7.41
Calcium (%)	0.33	0.09	0.20	0.55
Phosphorus (%)	0.55	0.04	0.48	0.61
Phytic phosphorus (%)	0.36	0.03	0.29	0.41
Ureatic activity (ΔpH)	0.046	0.030	0.01	0.10
Protein solubility (%)	81.74	3.220	75.12	86.24
Soluble protein (%)	32.66	1.40	30.22	35.31
EAS^1)^ (Mcal/kg)	3.31	1.14	0.63	5.52
Gross energy (kcal/kg)	5,826	46.76	5,762	5,914
DE (kcal/kg)	4,558	161.23	4,248	4,733
ME (kcal/kg)	4,457	186.09	4,070	4,721
MEn (kcal/kg)	4,344	172.45	3,983	4,610

SD, standard deviation; DE, digestible energy; ME, metabolizable energy; ME_n_, ME corrected for nitrogen balance.

1) EAS, energy applied per kg of sample (EAS = SH×TIM×TPT/1,000,000); SH, specific heat; TIM, processing time; TPT, processing temperature.

**Table 5:** Effect of partial removal of the hull on the composition, digestibility coefficients and energy values of full-fat deactivated soybeans (dry matter basis)

Variable	Unpeeled (n = 7)	Partially dehulled (n = 7)	Pr>|t|
Crude protein (%)	39.78±0.29	40.15±0.51	0.545
Ether extract (%)	24.57±0.39	24.40±0.61	0.822
Ash (%)	5.335±0.045	5.296±0.088	0.697
Crude fiber (%)	5.157±0.280	3.896±0.372	0.019
Acid detergent fiber (%)	6.284±0.195	4.391±0.359	0.0006
Neutral detergent fiber (%)	11.49±0.49	9.348±0.682	0.025
Hemicellulose (%)	5.204±0.458	4.957±0.563	0.740
Calcium (%)	0.324±0.039	0.328±0.035	0.942
Phosphorus (%)	0.522±0.013	0.569±0.012	0.021
Phytic phosphorus (%)	0.375±0.010	0.352±0.015	0.226
Ureatic activity (ΔpH)	0.049±0.012	0.044±0.012	0.803
Protein solubility (%)	81.75±1.11	81.73±1.41	0.992
Soluble protein (%)	32.51±0.40	32.81±0.66	0.710
Gross energy (kcal/kg)	5,813±19	5,840±16	0.295
CADDM (%)	80.71±0.89	82.58±0.76	0.135
CADCP (%)	85.74±1.44	87.56±1.57	0.410
DE (kcal/kg)	4,506±65	4,610±54	0.242
ME (kcal/kg)	4,429±83	4,484±59	0.602
MEn (kcal/kg)	4,320±80	4,369±51	0.618

CADDM, coefficient of apparent digestibility of dry matter; CADCP, coefficient of apparent digestibility of crude protein; DE, digestible energy; ME, metabolizable energy; ME_n_, ME corrected for nitrogen balance.

**Table 6:** Descriptive statistics of amino acid content of full-fat deactivated soybean samples (dry matter basis)

Amino acid (%)	Mean (n = 14)	SD	Minimum	Maximum	CV
Essential amino acids					
Arginine	2.919	0.122	2.72	3.07	4.18
Histidine	1.088	0.050	0.98	1.14	4.60
Isoleucine	1.836	0.149	1.62	2.01	8.12
Leucine	3.145	0.121	2.98	3.29	3.85
Lysine	2.625	0.085	2.47	2.75	3.24
Methionine	0.484	0.037	0.43	0.57	7.64
Phenylalanine	2.159	0.115	1.97	2.29	5.33
Threonine	1.714	0.051	1.62	1.81	2.98
Tryptophan	0.661	0.042	0.60	0.73	6.35
Valine	1.889	0.099	1.74	2.04	5.24
Nonessential amino acids					
Alanine	1.862	0.056	1.77	1.96	3.01
Aspartic acid	4.640	0.194	4.25	5.03	4.18
Cystine	0.660	0.084	0.55	0.81	12.73
Glutamic acid	7.479	0.401	6.89	8.12	5.36
Glycine	1.776	0.033	1.73	1.85	1.86
Hydroxyproline	0.069	0.016	0.03	0.09	23.19
Proline	2.077	0.089	1.95	2.22	4.29
Serine	2.212	0.068	2.09	2.32	3.07
Tyrosine	1.464	0.112	1.27	1.58	7.65
Total amino acids	40.63	1.647	36.88	42.70	4.05

SD, standard deviation; CV, coefficient of variation.

**Table 7:** Effect of partial removal of the hull on the amino acid content of full-fat deactivated soybeans (dry matter basis)

Amino acid (%)	Unpeeled (n = 7)	Partially dehulled (n = 7)	Pr>|t|
Essential amino acids			
Arginine	2.877±0.047	2.961±0.043	0.207
Histidine	1.081±0.018	1.094±0.020	0.648
Isoleucine	1.836±0.053	1.836±0.064	1.000
Leucine	3.114±0.043	3.176±0.048	0.363
Lysine	2.633±0.025	2.617±0.040	0.744
Methionine	0.470±0.012	0.497±0.015	0.178
Phenylalanine	2.154±0.038	2.163±0.051	0.896
Threonine	1.689±0.020	1.739±0.015	0.065
Tryptophan	0.649±0.014	0.674±0.017	0.271
Valine	1.880±0.034	1.899±0.043	0.740
Nonessential amino acids			
Alanine	1.833±0.018	1.891±0.019	0.047
Aspartic acid	4.623±0.094	4.657±0.052	0.755
Cystine	0.651±0.029	0.669±0.036	0.717
Glutamic acid	7.346±0.131	7.611±0.164	0.229
Glycine	1.767±0.009	1.784±0.016	0.355
Hydroxyproline	0.077±0.004	0.061±0.007	0.070
Proline	2.043±0.027	2.111±0.036	0.155
Serine	2.203±0.020	2.221±0.032	0.630
Tyrosine	1.484±0.036	1.444±0.050	0.527
Total amino acids	40.41±0.43	40.85±0.80	0.638

**Table 8:** Correlation coefficients of energy values with chemical composition, quality indicators and processing parameters of full-fat deactivated soybean samples (dry matter basis)

Items	GE	DE	ME	ME_n_
GE (kcal/kg)	1.00	−0.35	−0.26	−0.26
DE (kcal/kg)	−0.35	1.00	0.88^*^	0.87^*^
ME (kcal/kg)	−0.26	0.88^*^	1.00	0.99
MEn (kcal/kg)	−0.26	0.87^*^	1.00	1.00
Crude protein (%)	0.37	−0.42	−0.41	−0.39
Ether extract (%)	0.07	−0.26	−0.24	−0.22
Ash (%)	0.14	0.01	0.40	0.38
Crude fiber (%)	0.19	−0.65^*^	−0.36	−0.33
Acid detergent fiber (%)	−0.24	−0.33	−0.07	0.04
Neutral detergent fiber (%)	−0.11	−0.37	−0.006	−0.08
Hemicellulose (%)	−0.24	−0.12	−0.07	0.13
Calcium (%)	0.19	−0.14	0.03	0.02
Phosphorus (%)	0.08	0.54^*^	0.53^*^	0.56^*^
Phytic phosphorus (%)	−0.29	−0.42	0.44	−0.42
Ureatic activity (ΔpH)	0.06	−0.32	0.33	−0.35
Protein solubility (%)	−0.06	0.28	0.55^*^	0.61^*^
Soluble protein (%)	0.15	−0.01	0.24	0.31
Processing time (s)	−0.42	−0.19	−0.26	−0.22
Processing temperature (°C)	0.37	−0.38	−0.33	−0.32
Processing pressure (kgf/cm^2^)	−0.17	−0.53^*^	−0.59^*^	−0.57^*^
EAS^1)^ (Mcal/kg)	−0.35	−0.25	−0.31	−0.26

GE, gross energy; DE, digestible energy; ME, metabolizable energy; ME_n_, ME corrected for nitrogen balance.

1) EAS, energy applied per kg of sample (EAS = SH×TIM×TPT/1,000,000); SH, specific heat; TIM, processing time; TPT, processing temperature.

* p<0.05.

**Table 9:** Selected prediction equations for digestible energy (DE) and respective AIC values, coefficients of determination (R^2^), prediction errors, and ureatic activity for maximum DE value, generated from the chemical composition, quality indicators, and processing parameters of 14 samples of full-fat deactivated soybeans (dry matter basis)

N°	Equation	AIC	R^2^	Prediction error (kcal)				Relative prediction error (%)				UA for maximum DE (ΔpH)
	
				Mean	SD	Min	Max	Mean	SD	Min	Max	
1	Y = 4,045.0−48.2CF+8,962.9UA− 98,096.6UA^2^+1,117.0P	166.24	0.85	45.98	39.56	3.16	131.12	1.01	0.86	0.067	2.81	0.046
2	Y = 2,670.2−41.9CF+10,364.0UA− 127,507.0UA^2^+49.5CP	167.51	0.84	50.72	37.88	6.97	116.34	1.11	0.83	0.16	2.63	0.041
3	Y = 4,156.8+939.2P−22.8NDF+10,159.4UA− 114,950.0UA^2^	167.74	0.84	49.88	39.95	6.64	132.22	1.09	0.89	0.15	2.99	0.044
4	Y = 4,719.9−54.8CF+8,018.9UA−95,188.1UA^2^	168.74	0.80	53.72	46.78	1.75	127.44	1.18	1.03	0.039	2.88	0.042
5	Y = 4,474.9+8,898.7UA−109,857.0UA^2^	172.56	0.69	68.90	53.99	18.71	233.97	1.52	1.22	0.44	5.29	0.041
6	Y = 5,002.3−98.2CF	179.43	0.42	95.03	73.47	0.89	238.60	2.10	1.62	0.020	5.05	-

AIC, Akaike information criterion; SD, standard deviation; Min, minimum; Max, maximum; UA, ureatic activity; DE, digestible energy; Y, estimated DE; CF, crude fiber; UA^2^, ureatic activity quadratic component; P, phosphorus; CP, crude protein; NDF, neutral detergent fiber.

**Table 10:** Selected prediction equations for metabolizable energy (ME) and respective AIC values, coefficients of determination (R^2^), prediction errors, and ureatic activity for maximum ME value, generated from the chemical composition, quality indicators, and processing parameters of 14 samples of full-fat deactivated soybeans (dry matter basis)

N°	Equation	AIC	R^2^	Prediction error (kcal)				Relative prediction error (%)				UA for maximum ME (ΔpH)
	
				Mean	SD	Min	Max	Mean	SD	Min	Max	
1	Y = 3,060.2+16.3PS−443.8PP+11,516.7UA−104,776.0UA^2^ +93.1EAS^1)^	176.69	0.80	56.62	56.51	0.79	207.82	1.34	1.31	0.02	4.93	0.055
2	Y = 1,231.7+15.0PS+9,730.0UA−107,594.0UA^2^ +399.9ASH−58.8CF	178.59	0.77	72.21	49.29	15.76	175.68	1.62	1.10	0.35	3.79	0.045
3	Y = 2,676.3+12,716.7UA−139,023.0UA^2^+325.3ASH−131.6PP	178.12	0.74	72.23	57.93	0.75	158.28	1.64	1.33	0.02	3.68	0.046
4	Y = 1,988.9+11,979.4UA−136,049.0UA^2^+470.2ASH−43.7CF	178.75	0.73	74.05	59.00	3.79	166.40	1.66	1.31	0.08	3.64	0.044
5	Y = 2,166.4+13,479.3UA−154,864.0UA^2^+396.5ASH	179.02	0.68	75.66	69.81	8.14	244.54	1.71	1.59	0.18	5.67	0.044
6	Y = 4,269.5+13,742.2UA−157,576.0UA^2^	182.27	0.54	106.32	62.36	2.11	254.05	2.38	1.44	0.05	5.89	0.044
7	Y = 4,735.0+266.8PP	185.06	0.35	124.03	77.78	32.49	277.82	2.80	1.79	0.72	6.59	-
8	Y = 1,854.8+31.8PS	185.96	0.30	127.49	81.35	11.06	282.48	2.90	1.97	0.25	6.70	-

AIC, Akaike information criterion; SD, standard deviation; Min, minimum; Max, maximum; UA, ureatic activity; Y, estimated ME; PS, protein solubility; PP, processing pressure; UA^2^, ureatic activity quadratic component; CF, crude fiber.

1) EAS, energy applied per kg of sample (EAS = SH×TIM×TPT/1,000,000); SH, specific heat; TIM, processing time; TPT, processing temperature.

**Table 11:** Selected prediction equations for metabolizable energy corrected for nitrogen balance (ME_n_) and respective AIC values, coefficients of determination (R^2^), prediction errors, and ureatic activity for maximum ME_n_ value, generated from the chemical composition, quality indicators, and processing parameters of 14 samples of full-fat deactivated soybeans (dry matter basis)

N°	Equation	AIC	R^2^	Prediction error (kcal)				Relative prediction error (%)				UA for maximum ME_n_ (ΔpH)
	
				Mean	SD	Min	Max	Mean	SD	Min	Max	
1	Y = 2,789.8+18.1PS−431.5PP+10,240.6UA−92,301.3UA^2^ + 98.1EAS^1)^	171.21	0.84	44.21	51.41	0.52	168.75	1.02	1.20	0.011	4.06	0.055
2	Y = 1,277.3+18.1PS+8,404.5UA−94,031.1UA^2^+319.34ASH−51.0CF	176.11	0.77	62.68	50.06	3.49	154.35	1.44	1.13	0.081	3.43	0.045
3	Y = 2,691.8+20.5PS−164.3PP+9,010.3UA− 93,573.4UA^2^	175.22	0.75	68.03	48.08	6.55	175.80	1.58	1.14	0.14	4.23	0.048
4	Y = 1,787.0+11.6PS+10,907.7UA−125,536.0UA^2^+274.9ASH	177.69	0.71	71.63	56.35	10.46	215.99	1.65	1.32	0.24	5.11	0.043
5	Y = 2,326.0+12,247.5UA−142,526.0UA^2^+349.2ASH	177.10	0.68	78.80	54.20	17.44	207.73	1.82	1.26	0.41	4.91	0.043
6	Y = 2,692.2+18.5PS+10,266.9UA−117,047.0UA^2^	178.62	0.64	78.63	63.80	6.43	226.42	1.81	1.49	0.16	5.35	0.044
7	Y = 4,178.4+12,479.1UA−144,914.0UA^2^	179.86	0.54	98.60	55.24	6.18	216.11	2.26	1.27	0.15	5.11	0.043
8	Y = 1,688.2+32.5PS	182.47	0.37	110.89	74.48	14.69	239.17	2.59	1.81	0.34	6.00	-
9	Y = 4,594.2−239.7PP	183.37	0.33	117.96	71.10	12.89	236.52	2.73	1.67	0.29	5.68	-

AIC, Akaike information criterion; SD, standard deviation; Min, minimum; Max, maximum; UA, ureatic activity; Y, estimated ME_n_; PS, protein solubility; PP, processing pressure; UA^2^, ureatic activity quadratic component; CF, crude fiber.

1) EAS, energy applied per kg of sample (EAS = SH×TIM×TPT/1,000,000); SH, specific heat; TIM, processing time; TPT, processing temperature.

**Table 12:** p-values from the maximum likelihood parameter estimates analysis for each variable contemplated in the prediction equations for digestible energy, metabolizable energy, and metabolizable energy corrected for nitrogen balance

Equation	Variables									

	CF	UA	UA^2^	P	CP	NDF	PS	PP	EAS^1)^	ASH
Digestible energy										
Y = 4,045.0−48.2CF+8,962.9UA−98,096.6UA^2^+1,117.0P	0.006	0.001	0.001	0.021	-	-	-	-	-	-
Y = 2,670.2−41.9CF+10,364.0UA−127,507.0UA^2^+49.5CP	0.031	0.001	0.001	-	0.057	-	-	-	-	-
Y = 4,156.8+939.2P−22.8NDF+10,159.4UA−114,950.0UA^2^	-	0.001	0.001	0.078	-	0.022	-	-	-	-
Y = 4,719.9−54.8CF+8,018.9UA−95,188.1UA^2^	0.007	0.004	0.001	-	-	-	-	-	-	-
Y = 4,474.9+8,898.7UA−109,857.0UA^2^	-	0.009	0.001	-	-	-	-	-	-	-
Y = 5,002.3−98.2CF	0.002	-	-	-	-	-	-	-	-	-
Metabolizable energy										
Y = 3,060.2+16.3PS−443.8PP+11,516.7UA−104,776.0UA^2^+93.1EAS	-	0.001	0.001	-	-	-	0.040	0.001	0.032	-
Y = 1,231.7+15.0PS+9,730.0UA−107,594.0UA^2^+399.9ASH−58.8CF	0.033	0.008	0.005	-	-	-	0.127	-	-	0.007
Y = 2,676.3+12,716.7UA−139,023.0UA^2^+325.3ASH−131.6PP	-	0.001	0.001	-	-	-	-	0.073	-	0.027
Y = 1,988.9+11,979.4UA−136,049.0UA^2^+470.2ASH−43.7CF	0.117	0.001	0.001	-	-	-	-	-	-	0.002
Y = 2,166.4+13,479.3UA−154,864.0UA^2^+396.5ASH	-	0.001	0.001	-	-	-	-	-	-	0.012
Y = 4,269.5+13,742.2UA−157,576.0UA^2^	-	0.003	0.001	-	-	-	-	-	-	-
Y = 4,735.0−266.8PP	-	-	-	-	-	-	-	0.006	-	-
Y = 1,854.8+31.8PS	-	-	-	-	-	-	0.014	-	-	-
Metabolizable energy corrected for nitrogen balance										
Y = 2,789.8+18.1PS−431.5PP+10,240.6UA−92,301.3UA^2^+98.1EAS	-	0.001	0.001	-	-	-	0.006	0.001	0.006	-
Y = 1,277.3+18.1PS+8,404.5UA−94,031.1UA^2^+319.34ASH−51.0CF	0.044	0.013	0.007	-	-	-	0.043	--		0.019
Y = 2,691.8+20.5PS−164.3PP+9,010.3UA−93,573.4UA^2^	-	0.006	0.005	-	-	-	0.011	0.010	-	-
Y = 1,787.0+11.6PS+10,907.7UA−125,536.0UA^2^+274.9ASH	-	0.002	0.001	-	-	-	0.222	-	-	0.071
Y = 2,326.0+12,247.5UA−142,526.0UA^2^+349.2ASH	-	0.001	0.001	-	-	-	-	-	-	0.017
Y = 2,692.2+18.5PS+10,266.9UA−117,047.0UA^2^	-	0.009	0.003	-	-	-	0.056	-	-	-
Y = 4,178.4+12,479.1UA−144,914.0UA^2^	-	0.003	0.001	-	-	-	-	-	-	-
Y = 1,688.2+32.5PS	-	-	-	-	-	-	0.004	-	-	-
Y = 4,594.2−239.7PP	-	-	-	-	-	-	-	0.009	-	-

CF, crude fiber; UA, ureatic activity; UA^2^, ureatic activity quadratic component; P, phosphorus; CP, crude protein; NDF, neutral detergent fiber; PS, protein solubility; PP, processing pressure.

1) EAS, energy applied per kg of sample (EAS = SH×TIM×TPT/1,000,000); SH, specific heat; TIM, processing time; TPT, processing temperature.

## Data Availability

The data that support this study may be shared upon request to the first author if appropriate.
